# Editorial: “The dawn of future orthopaedic surgery: intraoperative navigation and robotic assistance”

**DOI:** 10.3389/fsurg.2023.1209454

**Published:** 2023-11-17

**Authors:** Junqing Lin, Hongyi Zhu

**Affiliations:** ^1^ Department of Orthopaedic Surgery, Shanghai Sixth People's Hospital, Shanghai Jiao Tong University School of Medicine, Shanghai, China; ^2^ National Center for Orthopaedics, Shanghai, China

**Keywords:** orthopaedic surgery, robotic surgical, robot-assisted surgery, robot-enhanced procedures, orthopaedic

**Editorial on Research Topic** “The dawn of future orthopaedic surgery: intraoperative navigation and robotic assistance”

Recently, the efficacies of many classic surgeries (1–3) have been greatly challenged by high-level evidence. In my understanding, every surgical procedure has two sides, yin and yang. For the yang side, the procedure could directly bring therapeutic effects to patients via invasive manipulation. In contrast, for the yin side, invasive procedures would inevitably cause Iatrogenic injury to patients as well. Technological innovation in orthopedics is still relatively slow. Since the emergence of the Da Vinci surgical robot, robotic minimally invasive surgery has been a hot topic pursued by various departments (4). Robotic orthopedic surgeries are a New Hope for musculoskeletal and sports medicine, just as the role of Luke Skywalker for the galaxy (5).

In recent years, many orthopedic surgical robots have emerged in the field of orthopedics for clinical applications, including MAKO (6) and NAVIO (7) surgical robots represented by joint replacement surgery, as well as MAZOR (8) surgical robots represented by spine surgery. The use of surgical robots in orthopedic procedures has improved precision and minimized invasiveness. Although these robots have injected new vitality into the improvement of orthopedic surgical techniques and enabled a certain degree of precision and efficacy enhancement, their application has not been widely used despite almost 40 years of development since their first use in orthopedics in 1986 (5). In contrast, the da Vinci surgical robot has been widely used in various fields since its first clinical use in 1997, owing to its high-resolution three-dimensional imaging system and precise mechanical arm controller. The da Vinci surgical robot can accurately achieve therapeutic goals under minimally invasive conditions, significantly improve efficacy, reduce the risk of postoperative complications, and shorten hospitalization time (9). These observations suggest that numerous deficiencies in orthopedic surgical robots have limited their development in the past decade.

The final acceptance of robotic surgery in general orthopedic surgery depends on its cost-effectiveness. In contrast to the da Vinci robotic system, the cost of robotic surgery is notably higher. Different types of prostheses and equipment may have different robots available, which further increases the cost of surgery. Moreover, there is currently no evidence that this increase in cost can bring benefits to patients sufficient enough to balance out this cost (10). Many orthopedic surgical robots require a lot of time to process data and locate before surgery, making the surgical procedure extremely complex, which leads to longer surgery times compared to traditional surgeries, and makes it very difficult for orthopedic surgical robots to be widely promoted (5). In addition, as orthopedic surgical robots are a product of recent years’ development, there is currently a lack of high-quality clinical research evidence on their long-term efficacy. Therefore, the academic community still has many doubts about their effectiveness in treatment. A prospective randomized controlled study conducted by Bell et al. demonstrated that the utilization of the MAKO robotic arm system for unicompartmental knee replacement (UKA) resulted in a significant improvement in surgical precision when compared to conventional surgical procedures (11). However, in contrast, another five-year clinical randomized controlled trial did not find a statistically significant difference in surgical accuracy for UKA between manual and robots (12). This finding aligns with a similar study that compared manual and robotic-assisted UKA. Intriguingly, the same surgeon exhibited higher surgical accuracy with manual surgery as opposed to robot-assisted surgery (13). As a result, there is an ongoing debate in the academic community regarding the potential benefits of using surgical robots for orthopedic surgeries and the precise advantages they may offer to patients.

So far, computer-assisted navigation technology has been widely used in orthopedic surgery, but its application varies among different sub-specialties. With the continuous maturity of technology, more and more joint replacement surgeries are assisted by robots for auxiliary operations. Since 2008, with the increasing trend of technology utilization and more robot systems entering the market, the possibility of using robots in orthopedic surgery is expected to increase (14). However, critical issues must be considered during the research and application process of these devices. It is essential to determine whether surgical robots can genuinely address clinical problems and enhance postoperative efficacy while reducing costs without compromising quality. Only by addressing these critical issues can we ensure that the innovation and progress of robotic devices in future orthopedic surgeries will continue to advance.
